# Real-Time Stream Data Anonymization via Dynamic Reconfiguration with *l*-Diversity-Enhanced SUHDSA

**DOI:** 10.3390/s26010095

**Published:** 2025-12-23

**Authors:** Jiyeon Lee, Soonseok Kim

**Affiliations:** Department of AI Information Security, Halla University, Wonju 26404, Republic of Korea; jiyeon.lee@halla.ac.kr

**Keywords:** *l*-diversity, *k*-anonymity, real-time data stream, stream anonymization, microaggregation, dynamic reconfiguration, information loss, privacy–utility trade-off, UBDSA, SUHDSA

## Abstract

Pipelines that satisfy *k*-anonymity alone remain vulnerable to attribute disclosure under skewed sensitive attributes. We studied real-time anonymization of high-throughput data streams under strict delay budgets (β). We jointly enforced *k*-anonymity and *l*-diversity via a delay-aware Monitor–Trigger–Repair controller that selects swap vs. merge by minimizing a weighted objective λΔIL + (1 − λ)ΔRT while bounding overhead with a neighbor cap (c) and a growth cap (γ). On UCI Adult stream replay, we identified operating regions where stricter privacy does not necessarily increase distortion: with moderate-to-high k and sufficiently large β, groups satisfy l preemptively, reducing reconfigurations and avoiding aggressive generalization, thereby mitigating information loss relative to *k*-only baselines. Privacy metrics (*l*-satisfaction rate and entropy) also improved. We further report a focused sensitivity analysis on λ, c, and γ and evaluate an entropy-driven adaptive $l_{t}$ controller, showing that these levers provide interpretable trade-offs between latency and distortion and can suppress excessive reconfiguration and tail latency.

## 1. Introduction

Real-time analytics increasingly operate on continuous data streams in healthcare, finance, and mobility, where sensitive attributes are common. Anonymization must therefore preserve analytical utility under strict delay and throughput constraints while preventing both reidentification and attribute disclosure. Traditional batch-oriented methods are ill suited to these constraints, so stream-native approaches are needed. However, existing *k*-anonymity-only stream methods do not explicitly enforce sensitive-attribute (SA) diversity under delay constraints, leaving a gap in real-time settings.

This study addresses the challenge of balancing three real-time objectives: security (low reidentification and attribute-disclosure risk), utility (low information loss (IL)), and performance (responsiveness). Classical privacy models such as *k*-anonymity [1], *l*-diversity [2], and *t*-closeness [3] provide principles for risk mitigation; however, enforcing them efficiently within streaming pipelines remains difficult because of limited buffering, non-stationary distributions, and the need for rapid grouping and transformation.

Recent work in stream anonymization has explored microaggregation, improved clustering and assignment, and delay–distortion co-optimization, demonstrating reduced IL while meeting delay targets. However, when SA distributions are skewed within equivalence classes, *k*-anonymity alone remains vulnerable to frequency and background-knowledge attacks. Such SA-skewed groups commonly arise in non-stationary streams with shifting class proportions.

We address the following problem: a high-throughput *k*-anonymity stream anonymizer (e.g., SUHDSA [4]) can meet performance goals yet still incur attribute-disclosure risk under SA skew. To resolve this, one must enforce sufficient SA diversity in real time without sacrificing throughput so that both reidentification and attribute-disclosure risks are mitigated under streaming constraints, particularly frequency and background-knowledge attacks in SA-skewed groups.

To that end, we extend SUHDSA with *l*-diversity-enhanced dynamic reconfiguration. The mechanism continuously monitors SA diversity during grouping and, upon violation, triggers lightweight merges/swaps before microaggregation. This on-the-fly correction of SA-skewed groups, followed by microaggregation to bound distortion, preserves real-time feasibility. High-throughput k-anonymity pipelines (e.g., SUHDSA) meet delay targets but remain vulnerable to attribute disclosure under SA skew, calling for a real-time approach that enforces both *k* and *l* without sacrificing throughput.

This work makes three contributions. First, on the methodological side, we integrate *l*-diversity verification and dynamic reconfiguration into a high-throughput *k*-anonymity streaming pipeline, enabling real-time maintenance of both *k*-anonymity and *l*-diversity under SA skew. Second, in the empirical evaluation of the UCI Adult dataset, the proposed method increases the runtime over SUHDSA by 3.94–7.87 s yet remains ~45.6% faster than the UBDSA; in utility, enforcing *l*-diversity increases IL at low *k* (up to +18% vs. SUHDSA) but reduces it as *k* increases (down to −19%), revealing operating regions where stronger privacy also reduces distortion. Third, for practice, we clarify that jointly tuning *k* and modest buffering *β* expands candidate pools, lowers reconfiguration frequency, and stabilizes utility at higher *k*, offering a practical privacy–utility trade-off for streaming applications.

This work departs from prior stream anonymization in three ways. First, we jointly enforce *k*-anonymity and *l*-diversity under an explicit delay budget β, casting privacy–utility–delay as an actionable triad. Second, we introduce a decision-theoretic controller that minimizes λΔIL + (1 − λ)ΔRT and constrains the overhead via caps (c, γ). Third, we provide deployment guidance (tuning β first, then *k*) and risk-based interpretation (*l*-satisfaction rate, entropy), turning *l*-diversity into a practical, real-time mechanism for streams.

The remainder of this paper is organized as follows. Section 2 reviews stream anonymization and privacy models relevant to real-time settings. Section 3 details the proposed mechanism and its integration with microaggregation. Section 4 presents the experimental setup, datasets, and metrics. Section 5 reports the results and discusses tuning guidance for *k* and *β*. Section 6 concludes with limitations and suggestions for future work.

## 2. Related Work

### 2.1. Taxonomy and Scope

We structure the prior work along four axes: (i) modeling primitives (sampling, clustering, microaggregation), (ii) privacy constraints (*k*-only vs. *k* + *l*), (iii) latency objectives (none, window-based, *β*-controlled), and (iv) system context (offline vs. streaming). Batch *k*-only methods lack delay control; some stream systems address throughput but omit *l*-diversity. Our method fills this gap by combining joint *k* + *l* under *β* with a controller-driven repair policy.

Research on anonymizing real-time data streams spans two lines: PPDM (interactive analytics over protected streams) and PPDP (publishing anonymized streams for downstream use) [5,6,7,8,9,10]. Within PPDM, interactive stream anonymization has been explored largely through local differential privacy [11,12,13,14,15,16,17] and private information retrieval [18]. Methods can be organized along four axes: privacy model (*k*-anonymity, *l*-diversity, *t*-closeness), grouping strategy (tree-based vs. clustering/microaggregation), delay control/objective (fixed deadline *δ* vs. joint delay–distortion optimization), and system context (stream engines and data modalities). This taxonomy clarifies how prior work balances utility, timeliness, and risk under streaming constraints and provides coordinates to situate our contribution.

### 2.2. PPDP Under Classical Models: Grouping and Delay Objectives

Early tree-based PPDP methods generalized QIDs hierarchically for streams [19,20], but clustering/microaggregation approaches became dominant because of better delay–utility trade-offs under a maximum delay *δ* [5,14,21,22,23,24]. A key milestone is UBDSA [25], which moved beyond “minimize IL under *δ*” [19,20,23,24,26,27,28,29,30,31,32,33,34,35,36,37,38] to jointly optimize average delay and distortion, introduced CAIL (Context-Aware IL), and formalized how δ shifts the frontier (higher *δ* → lower distortion but greater delay). Here, CAIL is an information-loss function designed to fairly aggregate and compare per-attribute distortion by handling numeric and categorical QIDs in a unified way—using taxonomy-aware distances (generalization trees) for categoricals and normalized scaling for numerics. With CAIL in place, grouping, microaggregation, and reconfiguration decisions under a delay constraint (δ) can be made consistently against a quantitative criterion. SUHDSA [4] leverages schema stability and repeated QID patterns to improve grouping/microaggregation efficiency, reporting faster runtime and lower distortion than UBDSA (e.g., up to 29.88 s faster and up to 77% lower distortion under comparable settings). Other works (e.g., Nasab & Ghaffarian [39]) improve the average delay against UBDSA [25] but do not chart the delay–distortion frontier via *δ* tuning, whereas SUHDSA explicitly shows an additional ≈4–5% distortion reduction by adjusting *δ*. At the system level, stream anonymization has been realized as drop-in modules for engines such as Apache Flink and as in-memory designs for high-volume flows [40,41,42]; modalities have extended beyond structured tables to IoT telemetry, social networking, location, and text streams [43,44,45,46]. Despite these advances, existing stream PPDP pipelines typically enforce *k*-anonymity only under delay constraints, leaving attribute-disclosure risk under SA skew.

### 2.3. Diversity-Aware Protection and the Open Gap

Whereas *l*-diversity (and related *t*-closeness) mitigates attribute disclosure by requiring multiple SA values per equivalence class [2], most stream PPDP pipelines enforce *k*-anonymity only under tight delay. In non-stationary streams, SA distributions frequently skew within groups, leaving *k*-only methods vulnerable to frequency and background-knowledge attacks even when *k* is satisfied. Enforcing SA diversity in real time is nontrivial: naïvely checking *l*-diversity per window increases the reconfiguration frequency and runtime, which conflicts with delay budgets and undermines responsiveness. Hence, the field lacks a diversity-aware, delay-conscious mechanism that preserves the throughput advantages of *k*-based pipelines while curbing attribute-disclosure risk under SA skew.

Within this taxonomy, our work operates in PPDP, adopts clustering/microaggregation, retains joint delay–utility awareness in the UBDSA/SUHDSA lineage, and contributes an *l*-diversity-enhanced dynamic reconfiguration that monitors SA diversity during grouping and triggers lightweight merge/swap before microaggregation when violations occur. This design preserves real-time feasibility (competitive runtime relative to SUHDSA and ~45.6% faster than UBDSA in our evaluation) while reducing attribute-disclosure risk under SA skew.

## 3. Proposed Data Anonymization Method

We augment SUHDSA with a real-time *l*-diversity enforcement mechanism so that, during grouping, every released group satisfies *k*-anonymity and *l*-diversity under a strict delay budget. The design directly targets the attribute-disclosure risk that arises when sensitive-attribute (SA) distributions are skewed inside *k*-anonymous groups while preserving the throughput and analytical utility of SUHDSA.

### 3.1. Setting, Notation, and System Constraints

Let the input stream be $DS=\{t_{1},t_{2},\ldots \}$. Each tuple t contains a set of quasi-identifiers (QIDs) $X=(X_{1},\ldots ,X_{m})$ and one sensitive attribute S. We form groups by QID similarity and release a group only if two conditions hold simultaneously: (i) the group size ∣G∣≥k (i.e., at least k records share similar QIDs), and (ii) the number of distinct SA values in the group satisfies D(G)≥l. Here, ∣G∣ is the size of group G; G.S is the multiset of SAs in G; and D(G) is the count of distinct SA values. Equivalently, $D\left(G\right)=|uniq(G.S)|$.

Incoming tuples are stored in a first-in, first-out (FIFO) buffer of size *β*, which serves as the buffering/window capacity (i.e., the maximum number of tuples held concurrently). For delay comparison, we define the time-equivalent budget *Tβ* =β/r, where *r* is the average arrival rate (events/s) used in our simulation (i.e., the effective throughput). Grouping begins when |*Buf*| ≥ *β* or when the *β*-based time budget elapses (e.g., event–time window + trigger in Flink; scheduled punctuation in Kafka streams). Grouping begins when ∣Buf∣≥B or when the β-based time budget elapses (e.g., event-time window + trigger in Flink; scheduled punctuation in Kafka streams). Provisional groups are then formed using CAILDistance with taxonomy-aware lowest common ancestor (LCA) placement. Following UBDSA [25] and SUHDSA [4], similarity for provisional grouping is computed via CAILDistance and the generalization tree’s common-ancestor rule: A new record is assigned to the cluster with the minimum CAILDistance, and when possible, QIDs that share a common ancestor in the taxonomy are placed together to reduce IL [4,25]. In this work, d(⋅,⋅) denotes CAILDistance, which is a mixed-type (numeric/categorical) distance between *QID* vectors. Numeric *QIDs* use per-dimensional normalization (e.g., [0, 1] scaling or z scores) followed by a standard Euclidean-/Manhattan-type distance; categorical *QIDs* use taxonomy-based distances derived from the depth of the lowest common ancestor (LCA) between categories. Optional per-dimension weights $w_{j}$ can emphasize important *QIDs*. The overall CAILDistance is the (weighted) sum across dimensions:

(1) $$d(QID(u),QID(v))=\sum_{j=1}^{m}w_{j}\;\delta_{j}(u_{j},v_{j}),$$

where $\delta_{j}$ is a normalized numeric distance if the j-th *QID* is numeric and an LCA-based categorical distance if it is categorical. The group representative rep(G) is defined per dimension as the mean or median for numeric *QIDs* and the mode or an appropriate higher taxonomy level (e.g., LCA) for categorical *QIDs*. Because rep(G) approximates the direction of microaggregation, a tuple whose *QID* is closer to rep(G) is more likely to join with smaller additional generalizations and lower IL increases.

Unlike *k*-only pipelines, we monitor SA diversity during grouping and repair any violations before microaggregation. Utility is measured by postaggregation IL, and performance is measured by runtime and delay. In our experiments, the *QIDs* are fixed to education, occupation, and the native country, and the SA is income. The *k*-anonymity constraint is enforced on the joint *QID* tuple (“education,” “occupation,” “native-country”), and *l*-diversity is enforced on “income.” We continue to use CAILDistance and the taxonomy’s common-ancestor structure for cluster assignment so that new records join the cluster that minimizes IL.

We use *β* for the per-window delay budget (maximum number of tuples buffered concurrently), *k* for the anonymity level, and *l* for the target diversity level. The controller objective is weighted by λ ∈ [0, 1] (larger λ prioritizes lower distortion), while *c* is the neighbor cap (top-*c* candidates), and *γ* is the merge growth cap enforcing ∣G∪H∣≤γk. We denote the reconfiguration frequency by *ρ* (merges + swaps per window·s). When enabled, $l_{t}$ denotes the entropy-driven adaptive diversity target, with a floor $l_{t}\geq l$. We denote the runtime by RT; ΔIL and ΔRT represent the marginal increases caused by a candidate reconfiguration action (swap/merge) relative to the current group state. For non-stationary streams, $l_{t}$ denotes the entropy-driven adaptive diversity target, and ρ denotes the reconfiguration frequency (merges + swaps per window·s).

The proposed pipeline operates window by window under the buffering budget *β*: It first forms provisional *k*-anonymous groups using QID similarity (CAILDistance/LCA placement) and then enforces *l*-diversity on the sensitive attribute (income) only when a group violates D(G)≥l. Repairs are lightweight and local: For a violating group, we inspect only the top-*c* QID-nearest neighbor groups and apply either (i) a minimal 1:1 swap that restores diversity while inducing the smallest combined increase in information loss and processing cost or (ii) a bounded merge (capped by ∣G∪H∣≤γ⋅k) when no feasible swap exists before microaggregation releases the group. The reconfiguration counter *ρ* simply records how many swap/merge actions were taken in the window and is later used to interpret sensitivity in latency/throughput. For example, consider β=6, k=3, and l=2 (binary income), where two provisional groups are formed: $G_{1}=\{\leq 50\;K,\leq 50K,\;\leq 50\;K\}$ and $G_{2}=\{\leq 50\;K,>50\;K,>50\;K\}$. Since $D(G_{1})=1<2$, $G_{1}$ violates l-diversity, the algorithm therefore selects a QID-nearest record pair across $G_{1}$ and $G_{2}$ and performs a swap, yielding $G_{1}^{\prime }=\{\leq 50\;K,\leq 50\;K,>50\;K\}$ and $G_{2}^{\prime }=\{\leq 50\;K,>50\;K,>50\;K\}$, so that both groups satisfy the k and l constraints. If no feasible swap exists, $G_{1}$ is merged with its nearest feasible neighbor under the γ⋅k size cap, and the resulting group is then microaggregated.

### 3.2. l-Diversity-Enhanced Dynamic Reconfiguration (Monitor–Trigger–Repair)

For each provisional group G, we compute D(G) and perform reconfiguration according to a monitor–trigger–repair pattern—monitor for violations, trigger when a violation is detected, and immediately repair via swap/merge. If D(G)<l, we mark G for reconfiguration with priority τ(G)=l−D(G). When priorities tie, we break ties by (i) increasing group age (to respect delay), (ii) decreasing the remaining time to the β deadline, and (iii) increasing ∣G∣ to reduce the likelihood of later merges. The reconfiguration proceeds lightest-first. A swap exchanges a small number of tuples between G and its QID-nearest neighbor H, provided that both groups remain feasible afterward (∣G∣, ∣H∣≥k and D(G),D(H)≥l). Swapping is guided by similarity to the other group’s representative (mean/median for numeric QIDs; mode/taxonomy level for categorical QIDs). If a swap cannot satisfy both constraints, we perform a merge with a QID-nearest feasible neighbor, capping the merged size to avoid excessive growth.

To limit the overhead, the neighbor search is restricted to the top c=3 nearest groups. We choose between swap and merge by minimizing a weighted combination of the expected information-loss increase and runtime increase as follows:

choose action = argmin[λΔIL + (1 − λ)ΔRT](2)

where λ∈[0, 1] trades off ΔIL and ΔRT (a larger λ favors utility preservation). Here, ΔIL denotes the increase in IL after applying a candidate action (swap/merge) relative to the pre-action state of the affected group(s), and ΔRT denotes the corresponding additional processing time attributed to executing that action in the same implementation. Unless stated otherwise, we use λ = 0.7 and *c* = 3 as defaults. For merges, we introduce a growth cap γ and enforce ∣G∪H∣≤γk so that the merged group does not become too large (γ=3 by default). Swaps use the minimum number of exchanges needed to restore D(G)≥l. Specifically, among all feasible 1:1 pairs $\left(x\in G,\;y\in H\right)$, we select the pair that minimizes

(3) d(QID(x),rep(G))+d(QID(y),rep(H))

and we then choose the repair action (swap vs. merge) by minimizing the objective λΔIL+(1−λ)ΔRT in (1) under the neighbor cap c and the growth cap γ. This criterion encourages each tuple to “fit” the other group’s representative with minimal structural disturbance and lower IL. The final choice must satisfy the feasibility constraints—after swap/merge, ∣G∣,∣H∣≥k, and D(G),D(H)≥l. Ties are resolved by the priority rules (group age, time to the β deadline, ∣G∣). If desired, a symmetric variant that also considers

(4) d(QID(x),rep(H))+d(QID(y),rep(G))

can be used, but in our implementation, the combination of the top-c neighbor restriction and the growth cap γ provides a lightweight search that achieves strong performance and quality when combined with (2).

**Figure 1:**
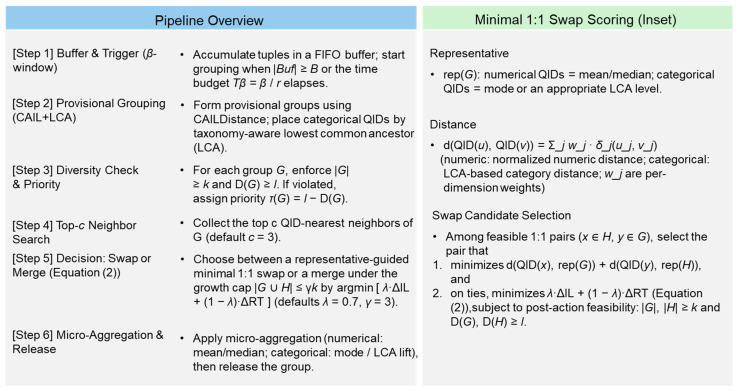
Real-time k/l-aware anonymization pipeline and the representative-guided swap rule. The left pipeline overview panel shows a β-triggered batching process (time budget $T_{\beta }=\beta /r$), provisional grouping via CAILDistance with taxonomy-aware LCA placement, and subsequent checks enforcing ∣G∣≥k and D(G)≥l. Violating groups receive priority τ(G)=l−D(G) and are repaired by inspecting only the top c neighbors and selecting a swap/merge that minimizes λΔIL+(1−λ)ΔRT (with merges bounded by ∣G∪H∣≤γk); groups are then microaggregated and released. The right representative-guided minimal swap panel summarizes the rule whereby, for a violating group G and neighbor H, we compute rep(G) and rep(H) and choose, among feasible 1:1 exchanges that keep ∣G∣,∣H∣≥k and D(G),D(H)≥l, the pair minimizing d(QID(x),rep(G))+d(QID(y),rep(H)) (numeric QIDs: normalized distance; categorical QIDs: LCA-based distance); ties are broken by the smaller λΔIL+(1−λ)ΔRT. If no feasible swap exists, we merge under the constraint ∣G∪H∣≤γk.

After reconfiguration, we apply microaggregation to the QIDs (numeric: mean/median; categorical: mode or taxonomy lift to LCA) and release the anonymized groups. The pseudocode of the proposed method appears in Algorithm 1, while Figure 1 complements it with an end-to-end walkthrough (CAILDistance + LCA grouping, representative-guided 1:1 swaps under (1), and microaggregation). The runtime-integration note after Algorithm 1 outlines how the pipeline can be mapped to Flink/Kafka streams. For readability, the detailed pseudocode of the adaptive $l_{t}$ controller is provided in Appendix B).
**Algorithm 1** *l*-diversity–enhanced dynamic reconfiguration (streaming SUHDSA)Input:  *DS*; *k*; *l*; *β*; distance *d*(·,·)            *λ* = 0.7  // weight between ΔIL and ΔRT (0–1)            *c* = 3     // number of QID-nearest neighbors inspected            *γ* = 3     // merge growth cap: |*G* ∪ *H*| ≤ γ·*k*             AdaptiveLControllerEnabled ∈ {true, false}  // default: false            // (optional) controller parameters when enabled:            *α* ∈ (0, 1]   // Exponential Moving Average (EMA) smoothing for *H_t*            HysteresisBand ∈ [0, 1], DwellWindows ∈ N Output: anonymized stream *A* 1    *Buf* ← ∅ 2    while *DS* not empty do 3           *t* ← ReadNextTuple(*DS*); *Buf* ← *Buf* ∪ {*t*} 4           if |*Buf*| ≥ *β* or time budget (*β*) elapsed then 5                  *ρ* ← 0 // reconfiguration counter for this window (swaps + merges); 6       // reset per window and logged to quantify repair overhead vs. latency/throughput 7                  *G_list* ← GroupByQID(*Buf, k, d*)             // provisional groups, |*G*| ≥ *k* 8                  // (optional) entropy-driven adaptive *l_t_* controller (see Appendix B) 9                 if AdaptiveLControllerEnabled then 10                *l_t_*_global ← AdaptiveLController(*Buf*, *l_min*, *l_max*, *α*, *HysteresisBand,* 11                                          *DwellWindows*, *Ĥ_*{*t−1*}) 12                else 13                       *l_t__global* ← *l* 14                end if 15                 for each *G* in *G_list* do 16                      // decide *l_t_*, for this step (global or per-group policy) 17                      *l_t_* ← (AdaptiveLControllerEnabled ? *l_t__global*:*l*) 18                      if *D*(*G*) = |uniq(*G.S*)| < *l_t_* then 19                             mark *G* with priority τ(*G*) = *l_t_*, − *D*(*G*) 20                      end if 21                end for 22                // Process marked groups by descending *τ*(*G*), then by age, *β*-deadline, |*G*| 23                for each marked G in DescendBy(*τ*(G), *age*(*G*), *time_to_β*(*G*), |*G*|) do 24                      *Cands* ← TopCNearestByQID(*G, G_list, c =* 3) 25                      *H* ← SelectFeasibleNeighbor(*Cands, k, l_t_*) 26                      // choose action by minimizing *λ*ΔIL + (1 − *λ*)ΔRT (Equation (1)) 27                      if SwapImprovesAndFeasible(*G, H, k, l_t_, λ*) then 28                          (*G, H*) ← SwapMinimalTuples(*G, H*) 29                          // minimal swap to restore *D*(·) ≥ *l_t_*
30                          *ρ* ← *ρ* + 1 // log: reconfiguration += 1 (used for Section 4.3 sensitivity analysis) 31                      else if MergeFeasible(*G, H, k, l_t_, γ* = 3) then 32                          *G* ← MergeGroups(*G, H*)                              // enforce |G ∪ H| ≤ γ·*k* 33                          *ρ* ← *ρ* + 1 // log: reconfiguration += 1 (used for Section 4.3 sensitivity analysis) 34                      end if 35                end for 36                for each *G* in *G_list* do 37                      *G.QID* ← MicroAggregate(*G.QID*) 38                      // numeric: mean/median; categorical: mode/taxonomy 39                end for 40                Output(*A, G_list*)                           // emit anonymized groups for this window 41                 LogWindowMetrics(*ρ*, p50/p95/p99 latency, throughput) 42                *Buf* ← ∅ 43           end if 44    end while 45    // Flush at stream end: if *DS* is empty but *Buf* is non-empty, 46    // finalize remaining records by forming feasible groups (|*G*| ≥ *k*, *D*(*G*) ≥ *l* or *l_t_*) 47    // or defer to the next window per *β* policy.

The proposed anonymization pipeline is designed to be compatible with real-time stream frameworks such as Apache Flink and Kafka Streams without modifying the core algorithm. In practice, (i) batches are formed by opening/closing windows (or triggers) according to the delay budget β, and (ii) the per-group state maintains representative values, sensitive-attribute histograms, priorities, and a shortlist of nearest neighbors. When reconfiguration is needed, the system inspects only the top-c nearest neighbors and chooses swap (1:1 exchange) or merge while jointly considering increases in IL and additional runtime. The implementation is straightforward: Flink provides event-time windows/triggers and stateful operators, whereas Kafka Streams provides state stores and scheduled punctuation. Both engines support exactly once semantics, ensuring safe, duplicate-free operation. The reported delays are interpreted against the time-equivalent budget β/r (with r as the mean arrival rate).

When selecting swap candidates, we evaluate how well the candidate records’ QIDs “fit” the other group’s representative—i.e., their proximity to that representative. A closer fit typically requires less additional generalization and therefore induces smaller increases in the IL. For numeric QIDs, we compute distances using standard metrics (e.g., Euclidean/Manhattan) after normalization; for categorical QIDs, we use the taxonomy’s LCA depth to measure between-category distances. Dimensions can be weighted if needed, and per-dimensional distances are aggregated to form the final score. Representatives are defined as the means/medians for numeric QIDs and modes (or a higher taxonomy level) for categorical QIDs. In short, swaps that better align with the target representatives tend to introduce less distortion.

Because the SA distribution can drift over time, a fixed l may become overly strict or overly lax. To address this, we provide an optional entropy-driven adaptive $l_{t}$ controller. It tracks the SA entropy via an exponential moving average and adjusts $l_{t}$ upward when entropy drops (skew increases) and downward when entropy is high (diversity is already sufficient). Lower/upper bounds, hysteresis, and a minimum dwell time prevent oscillations; a floor on $l_{t}$ preserves the intended risk bound, which we approximate as 1/k+1/l. Workload shifts can also change latency characteristics. During operation, β can be gently tuned using the target p95 latency: we adjust β in small steps, with hysteresis and a minimum dwell time, to stabilize the delay. The parameter l follows the adaptive controller above, and k can be varied slightly within a preset range to balance IL against the theoretical risk bound. For more aggressive optimization, a lightweight bandit/RL scheme could maximize a reward that reduces IL, keep latency within the budget, and lower the risk bound; however, such tuning introduces nontrivial overhead and is left for future work.

In summary, we preserve real-time responsiveness with a lightweight reconfiguration policy that inspects only the nearest neighbors and repairs them in small steps, selecting swaps/merges that align with group representatives to jointly control distortion and latency. When needed, an adaptive l and fine-grained adjustments of β and k reduce both over- and under-reconfiguration. This design integrates naturally with Flink and Kafka Streams and enables reliable performance tuning against the delay budget β/r.

### 3.3. End-to-End Pipeline, Decision Logic, and Guarantees

**Figure 2:**
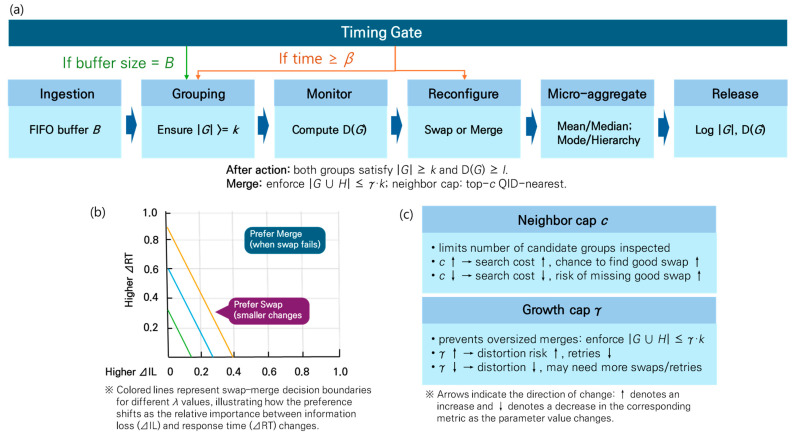
Real-time l-diversity-enhanced SUHDSA: (**a**) β-triggered windowing, provisional grouping via CAIL + LCA, *k*/*l* checks and swap/merge repair, then microaggregation and release. (**b**) Decision boundary from *λ*ΔIL + (1 − *λ*)ΔRT—higher *λ* enlarges the swap-favored region. (**c**) Qualitative effects of neighbor cap *c* and growth cap *γ* on search cost, distortion, and retries. Defaults: *λ* = 0.7, *c* = 3, *γ* = 3. (**a**) Timing gates determining when grouping and reconfiguration start under the β delay budget. (**b**) Decision boundary induced by *λ*ΔIL + (1 − *λ*)ΔRT; higher *λ* expands the swap-favored region as the controller prioritizes utility. (**c**) Effects of neighbor cap c and growth cap γ on search cost and distortion.

- End-to-end flow: Figure 2a (timing gates)

Tuples arrive at a FIFO buffer. When the buffer reaches size β or the β-based time limit expires $\left(T_{\beta }=\beta /r\right)$, grouping starts and enforces ∣G∣≥k. We then follow a monitor–trigger–repair pattern: detect violations of D(G)≥l and immediately correct them via swap or merge. Next, we perform microaggregation (numeric: mean/median; categorical: mode/LCA lift), release anonymized groups to the output stream, and advance the buffer to the next cycle. By correcting SA skew early, we reduce late-stage reshuffling and stabilize utility—an effect that is especially strong at larger k.

- Swap/merge choice: Figure 2b (decision boundary)

Reconfiguration actions are chosen by minimizing the weighted objective argmin[λΔIL + (1 − λ)ΔRT]. A larger λ prioritizes utility and enlarges the swap-favored region; a smaller λ favors merging to reduce latency/overhead. If the swap is infeasible, we fall back to merge under the growth cap ∣G∪H∣≤γk. The neighbor search is limited to the top-c nearest groups (defaults: λ = 0.7, c = 3, γ = 3).

- Search breadth and distortion control: Figure 2c (effects of c and γ)

The neighbor cap c trades off search cost against the chance of finding a good swap (smaller c: cost (↓)/hit-rate (↓); larger c: cost (↑)/hit-rate (↑)). The growth cap γ prevents oversized merges that spike distortion (γ larger: distortion risk (↑), retries (↓); γ smaller: distortion (↓), may require more swaps/retries). In practice, β is expanded first (within service-delay constraints) to enlarge the candidate pool, and then k is tuned to the target risk level.

- Parameter effects

Increasing β widens the within-window candidate pool, reduces the reconfiguration frequency, and improves the utility at higher k while increasing the delay. CAILDistance (taxonomy aware) uses mixed-type scaling and vectorized computation. The ablations validate the default settings of λ = 0.7, c = 3, and γ = 3.

- Privacy, consistency, and auditability (guarantees).

By construction, every released group satisfies ∣G∣≥k and D(G)≥l. Reconfiguration is permitted only if both affected groups remain feasible (∣G∣,∣H∣≥k and D(G),D(H)≥l); hence, the set of violating groups decreases monotonically, and the loop terminates. We log ∣G∣ and D(G) for every release to enable auditable compliance with k-anonymity and l-diversity (aligned with ISO/IEC 20889 [47]). For empirical privacy evaluation, we additionally compute (i) the l-satisfaction rate (LSR)—the percentage of released groups with D(G)≥l—and (ii) the per-group sensitive-attribute entropy H(SA∣G), which is summarized by the mean/median and minimum entropy value across released groups (i.e., the most vulnerable group).

## 4. Experiments, Complexity, and Results

### 4.1. Experimental Setup and Protocol

This study evaluates the proposed *l*-diversity–enhanced SUHDSA against SUHDSA [4] and UBDSA [25] with a focus on end-to-end runtime and IL across anonymity (*k*) and delay-budget (*β*) regimes. All the implementations share a common Python code base and were executed using Python 3.10 on Windows 11 with an Intel^®^ Core™ i9-12900KS (3.40 GHz; Intel Corporation, Santa Clara, CA, USA) and 64 GB of RAM. The experiments used the UCI ADULT (Census Income) dataset [48], with 32,561 tuples and 14 attributes (e.g., education, occupation, native country). For the SUHDSA baseline, we used our own reimplementation faithful to the cited paper [4] (not the authors’ original code). We validated parity by reproducing the key trends and ranges reported in [4] under matched (k,β) settings; any deviations are noted in Section 4.3. We fixed the QIDs to (education, occupation, native country) and enforced *k*-anonymity over this joint QID tuple. The SA is income, and to study the effect of diversity strength, we evaluated *l* ∈ {2, 3, 5} as follows: for l = 2, income is the standard binary label (≤50 K vs. >50 K); for l = 3, we discretized income into three brackets (≤30, (30, 60], >60); for l = 5, we used five brackets (≤20, (20, 40], (40, 60], (60, 80], >80). Because the original Adult dataset provides a binary income label, the multibracket income values for *l* ∈ {3,5} are synthetically assigned by stratified randomization, which is consistent with the dataset’s overall class proportions; the QID attributes were not altered. The buffering policy is FIFO; *β* denotes the maximum number of tuples buffered concurrently (FIFO), and it acts as a per-window delay budget in our replay setting (i.e., a larger *β* increases the maximum waiting capacity before release). All experiments were conducted using an optimized single-node Python implementation; we do not report results from a live Kafka/Flink deployment.

To ensure comparability with prior work, we applied UBDSA’s adaptive δ rule—which balances average delay and IL—identically to SUHDSA [4] and to the proposed method. We ran experiments over a grid of k∈{3,10,50} and β∈{10,50,100}, measuring the runtime and IL for each (k,β) configuration. To isolate the effect of l-diversity, we held (k,β) fixed and varied l∈{2,3,5} on a multiclass sensitive attribute; for each fixed (k,β), we recorded the average runtime and average IL. The UBDSA uses CAIL without l-diversity, and SUHDSA retains CAIL and β-aware fast aggregation without l-diversity. The proposed method preserves CAIL and the same δ adaptation while adding l-diversity verification and dynamic reconfiguration (swap/merge). A concise summary of the dataset, parameters, environment, and algorithmic features is provided in Table 1. All the quantitative results (including privacy metrics) are reported in Section 4.3. When analyzing the impact of l, we fixed k (over the QID tuple) and swept l∈{2,3,5} to observe its effect on runtime and IL.

Beyond runtime and IL, we also assessed how well privacy is actually preserved. We used two core metrics. First, the l-satisfaction rate (LSR) is the proportion of released groups that meet the target diversity level D(G)≥l; intuitively, it answers, in percentage terms, whether each group is “sufficiently mixed to avoid exposure.” Second, entropy quantifies the mixing balance of the sensitive attribute (e.g., income bracket) within each released group. We report the mean/median as the typical diversity and the minimum as the worst-case diversity, thereby capturing both the central tendency and the tail risk. Higher values of LSR and entropy indicate lower attribute-disclosure risk.

Three control quantities are central to the experimental setup: (i) β is the per-window delay budget, i.e., the maximum number of tuples that may reside concurrently in the FIFO buffer. A larger β enlarges the candidate pool, generally improving QID-similar grouping and reducing the need for reconfiguration; if β is too small, immediate release accelerates, but the system requires more reconfiguration to satisfy both k and l, which can increase the overhead. (ii) δ is the UBDSA-style adaptive threshold that automatically balances average delay against IL (applied uniformly to all methods for fairness). (iii) ρ is the reconfiguration frequency—the number of merges + swaps per window/second—which is a dynamic indicator of how often groups are adjusted. We analyzed ρ jointly with the latency distribution (p50/p95/p99) and throughput (events/s) to characterize the sensitivity of both average and tail (p99) latency, as well as capacity, to reconfiguration activity.

For orientation, the privacy and performance results are summarized in the following tables. Table 2 reports the runtime under varying k,β values; Table 3 reports the IL under the same conditions. Table 4 compares privacy metrics (LSR, H(SA∣G)) across methods, quantifying the benefit of enforcing l-diversity. Table 5 summarizes the l-sweep (average runtime and IL) at a fixed (k,β)=(10,50). Table 6 presents the robustness under severe SA imbalance (majority ratio ρ≈0.85). Table 7 reports the end-to-end (E2E) latency from stream replay (p50/p95/p99, throughput), including scenarios with synthetic network delay.

For each *(k, l, β)* configuration in Table 2 and Table 3, we repeated the stream replay experiment n=5 times using fixed random seeds and shared the same seeds across methods to enable paired comparisons under matched randomness. Unless stated otherwise, the results are reported as aggregated summaries over the n repetitions. Table 2 and Table 3 report mean ± standard deviation (SD) and 95% confidence intervals (CI); 95% CIs were computed using percentile bootstrap (B=10,000 resamples) over the repeated runs. For hypothesis testing, we used the Wilcoxon signed-rank test for paired comparisons (and the Mann–Whitney *U* test when a paired design was not applicable) and applied Bonferroni correction to report adjusted p-values.

### 4.2. Complexity

This section analyzes the computational and memory complexity of the proposed method. We begin with notations and assumptions. Let *β* denote the FIFO window size (i.e., the per-window delay budget/maximum number of tuples buffered concurrently). The number of QID attributes is m, and the required anonymity/diversity levels are k and l, respectively. The number of groups formed within a window is approximately g≃B/k. We write ν for the number of constraint-violating groups in a window (i.e., those failing ∣G∣≥k or D(G)≥l). During reconfiguration, we only search the top-c QID-nearest neighbors and cap merge growth by ∣G∪H∣≤γk. The UBDSA-style adaptive delay threshold δ is applied uniformly across all methods and affects the constants only.

The processing workflow is as follows. The B records arriving in a window are first grouped by QID similarity (categoricals are evaluated with a taxonomy/generalization tree; dimensional normalization is applied as needed). Each group is then checked against ∣G∣≥k and D(G)≥l. If a violation is detected, we attempt swap or merge reconfiguration, restricting the search to the top-c neighbors and enforcing the growth cap γ to prevent uncontrolled expansion. At the release time, ∣G∣ and D(G) are logged for auditability. From a security-model standpoint, the algorithm jointly enforces k-anonymity and l-diversity in accordance with ISO/IEC 20889 [47], thereby bounding reidentification and attribute-disclosure risks.

We now summarize the formal complexity. Stream ingestion touches each record once, resulting in O(n). Within a window, the QID-similar grouping is dominated by candidate comparison/placement, with the worst-case cost $O(B^{2}\;m)$. The l-diversity check is a linear scan over groups; since g≃B/k, this costs O(g⋅k)≈O(B). Reconfiguration occurs only for diversity violators but can dominate the cost envelope: without restrictions, mergers are ≈$O(g^{2})$ and swaps up to $O(g^{2}k)$. In practice, the top-c neighbor bound and the growth cap γ confine the effort so that the effective per-window reconfiguration cost is concentrated at approximately O(ν⋅c⋅k), where ν is the number of violating groups. Microaggregation is defined as O(B m). Accounting for windows in which reconfiguration is the bottleneck, the overall time bound is $T_{\mathrm{total}}=O(n\cdot B\cdot m)$, whereas the original SUHDSA (without reconfiguration) operates near O(nlog n). The space usage is dominated by buffered tuples and group metadata, $S_{\mathrm{total}}=O(B\cdot m),$ with only minor bookkeeping overhead beyond SUHDSA.

The parameter sensitivities can be summarized as follows. Increasing B(=β) increases the grouping cost but enlarges the candidate pool, typically reducing ν and thereby mitigating the reconfiguration cost; a practical balance point often emerges. A larger k tends to reduce ν by forming larger groups, but an overly large k can increase distortion and per-group cost. A higher l increases the likelihood of violations, increasing ν and the reconfiguration frequency. A larger neighbor cap c improves repair quality but adds O(νck) work, so a small default (e.g., c=3) is practical. The growth cap γ trades off ease of merging versus distortion risk (larger γ: fewer retries but greater risk of oversized groups; smaller γ: better distortion control but more swaps/retries). Finally, the swap–merge weight λ steers the decision toward minimizing ΔIL (utility-preserving, favoring swaps) or ΔRT (runtime-preserving, favoring merges).

Empirically, under typical streaming loads, the additional work from diversity checks and reconfiguration accounts for approximately 5–10% of the total runtime. In the same settings, the proposed method incurred an average +3.94–7.87 s overhead relative to SUHDSA while remaining approximately 45.6% faster than UBDSA, corroborating the above complexity analysis with observed performance and utility outcomes.

### 4.3. Results and Interpretation

As a sanity check for our SUHDSA reimplementation, we reproduced the characteristic timing and utility trends reported in [4] under matched (k,β) settings; the observed ranges in our runs fell within the expected bands. Starting with runtime, the proposed method incurs additional latency over SUHDSA due to *l*-diversity checks and the swap/merge search while still remaining markedly faster than UBDSA. Under $\left(k=3,\;\;\beta =100\right)$, the proposed method requires 12.30±2.10 s (min–max: 9.22–15.54), whereas SUHDSA requires 6.05±1.10 s (4.37–7.67), yielding an additional runtime of +4.85–7.87 s across the observed ranges (Table 2). When moving to $\left(k=50,\;\;\beta =100\right)$, the proposed method requires 10.05 ± 1.10 s (8.43–11.92) versus 5.95 ± 1.05 s (4.49–7.45) for SUHDSA, and the extra time decreases to +3.94–4.47 s. The reduction at larger k values is consistent with the intuition that greater class size naturally increases SA diversity within groups, thereby lowering the frequency of reconfiguration. Beyond these representative settings, we consistently observe sensitivity to β: A larger β enlarges the per-window candidate pool, stabilizes QID-similar grouping, and reduces reconfiguration needs, whereas a very small β accelerates immediate release but tends to trigger more repairs—and thus overhead—to satisfy both k and l. For statistical assessment, we repeated each setting n=5 times with matched random seeds and compared the proposed method vs. SUHDSA using the paired Wilcoxon signed-rank test with Bonferroni correction (Table 2).

With respect to IL, enforcing *l*-diversity introduces additional distortion relative to *k*-only anonymization; however, the magnitude of the IL overhead depends on the operating point. Under $\left(k=3,\;\;\beta =10\right)$, the proposed method reports IL=0.62±0.04  (min–max: 0.54–0.70), whereas SUHDSA reports 0.49±0.08  (0.36–0.61), corresponding to an absolute IL increase of 0.09–0.18 over SUHDSA across the observed ranges (Table 3). Under $\left(k=10,\;\;\beta =10\right)$, the propose method reports 0.64±0.06  (0.55–0.75) versus 0.58±0.11  (0.36–0.72) for SUHDSA, and the additional IL becomes smaller (0.03–0.19 over SUHDSA), indicating operating regions where improved grouping and SA balancing can mitigate the marginal distortion introduced by *l*-diversity enforcement even though the overhead remains positive. In general, IL tends to increase with k because stricter grouping increases pressure to generalize; however, a sufficiently large β improves matching among QID-similar tuples and moderates distortion even at higher k. In domains with skewed-SA distributions, modestly increasing β often yields better utility than indiscriminately increasing k. As in Table 2, all comparisons are based on n=5 repeated runs with matched seeds, and statistical significance for the proposed method vs. SUHDSA has been evaluated using the paired Wilcoxon signed-rank test with Bonferroni correction (Table 3).

To specifically assess the effect of *l*, we fixed *k* (applied to the QID tuple) and varied *l* ∈ {2,3,5}. As *l* increased, we observed (i) a moderate rise in runtime because of additional reconfiguration for diversity violations when *β* is tight and (ii) a reduction in IL under larger *k* or *β*, where larger candidate pools allow groups to meet *l*-diversity with fewer edits. These trends are consistent with those in Table 2 and Table 3 and are summarized for privacy metrics in Table 4. To quantify the privacy gains from enforcing *l*-diversity, we report two complementary measures: (i) the *l*-satisfaction rate (LSR), defined as the percentage of released groups with D(G)≥l, and (ii) the entropy of sensitive-attribute distributions within released groups, which is defined as $H\left(SA|G\right)$ (log base 2). We compared the LSR and the mean/min H(SA∣G) across methods under matched (k,β) configurations. A higher LSR indicates that a larger fraction of groups satisfies D(G)≥l, and higher entropy reflects more balanced SA distributions—both signaling reduced attribute-disclosure risk. As shown in Table 4, across all the matched (k,β) settings, the proposed method consistently attained a higher LSR and higher entropy than the k-only baselines (SUHDSA/UBDSA), with additional improvements as either k or β increased. For example, at (k,β)=(50,100), the proposed method achieved an LSR = 100% and a mean entropy of 0.90 bits, outperforming SUHDSA under the same conditions.

Holding (k,β)=(10,50) fixed, the proposed method showed a monotonic increase in both averages as l increased: average runtime 7.92→8.41→9.16 s for l=2,3,5 and average IL 47.50→52.00→64.50. By contrast, the *k*-only baselines were invariant to l: SUHDSA averaged 6.17 s/46.40, and UBDSA averaged 33.14 s/98.58. These results align with the expectation that stronger diversity constraints trigger more reconfiguration and induce additional distortion relative to *k*-only pipelines. Note that for l∈{3,5}, the multibracket income values were synthetically assigned (stratified randomization while keeping the global proportions) because the original Adult dataset provides a binary label. This synthetic assignment reduces the QID–SA correlation and can therefore make satisfying l-diversity somewhat easier than in real multiclass income settings where SA is more strongly correlated with QIDs, although increasing the SA cardinality itself (l=3→5) still raises the combinatorial difficulty. To partially compensate for this potential optimism, we additionally report the results on a skewed-SA subset (majority ratio ≈0.85), which represents an adverse mixing regime and inherently increases the likelihood of l-diversity violations.

To evaluate robustness under imbalanced SA distributions, we conducted a separate experiment on a subset in which the SA majority ratio was approximately 0.85 (i.e., ~85% of tuples share the same income bracket) while keeping the QID tuple unchanged. This setting inherently increases the likelihood of l-diversity violations and is therefore well suited to examining how the proposed swap/merge reconfiguration behaves under severe class imbalance. A summary appears in Table 6. Under an SA majority of ~0.85, the privacy of the k-only baselines degraded sharply (e.g., SUHDSA LSR ≈12%)), and the per-group SA entropy remained low, indicating elevated attribute-disclosure risk. By contrast, the proposed method sustained relatively high privacy even as l increased from 2 → 3 → 5 (LSR ≈92%→80%→62%; H(SA∣G) means ≈0.82→1.30→1.75 bits). As expected, these gains came with predictable costs: the average runtime increased (8.30 → 8.95 → 9.90 s), and IL increased (49.50 → 54.50 → 68.50). Notably, the minimum entropy under the proposed method remained strictly above zero, indicating the avoidance of single-value (highly vulnerable) groups, whereas the k-only baselines exhibited zero min-entropy in multiple configurations. In summary, even under severe SA skew, l-diversity-aware reconfiguration materially reduced the disclosure risk at a predictable runtime/utility cost, demonstrating that the method maintains a favorable privacy–utility balance under adverse distributional conditions.

We evaluated the end-to-end (E2E) delay by replaying the UCI Adult dataset as a stream. E2E delay is defined as the time from record ingress to sink emission of the anonymized group, including grouping and reconfiguration (swap/merge), while excluding physical network jitter (i.e., we measured processing time only). To approximate deployment effects, we also ran experiments with a synthetic network delay of δ∈{0,2,5} ms per record. We report the delay statistics as the median (p50) and upper percentiles p95 and p99, and we report the capacity as throughput (events/s). Unless stated otherwise, the replay is not rate-limited (the input stream is emitted as fast as possible), so the throughput reflects the achieved sink processing rate under each mode. We compute r as the total number of processed tuples divided by the wall-clock replay time (events/s). Here, p50 indicates that half of the events complete within that time, and p95/p99 indicate that 95%/99% of the events complete within the stated bound. In particular, p99 highlights tail latency under infrequent burst conditions. Empirically, even under high-skew workloads, p95 remained within the service-time target $T_{\beta }=\beta /r$ (Table 7), where r is the measured sink throughput (processing rate, events/s) for the corresponding mode. In other words, the vast majority (95%) of events met the target $T_{\beta }$, with only rare tails (p99) occasionally exceeding it.

We conducted experiments using the synthetic workload suite described in Section 4.1 (SA skew, bursty arrivals, and distribution drift) and report the quantitative results under a fixed delay budget β in Table 8 (runtime, IL, LSR, and entropy) and Table 9 (p50/p95/p99, throughput, and reconfiguration frequency ρ). Each condition was repeated n = 5 times with fixed random seeds. Across all stress scenarios, the proposed method consistently maintained higher diversity satisfaction (LSR) and higher SA entropy than the k-only baseline (Table 8), demonstrating robustness under non-stationery and stress conditions. In addition, as skew and burst intensity increased, we observed corresponding increases in the reconfiguration frequency ρ and tail latency (p99) (Table 9).

Taken together, the experiments show that the proposed method introduces an average of +3.94~7.87 s over SUHDSA while maintaining approximately 45.6% faster processing than UBDSA does. In terms of utility, distortion can be up to +18% greater in low-*k*, tight-*β* configurations but up to −19% lower as *k* increases or *β* widens, revealing privacy–utility sweet spots where enforcing *l*-diversity simultaneously strengthens protection and preserves—or even improves—data quality. A pragmatic tuning strategy is to increase *β* first within service-delay constraints to enlarge the candidate pool and stabilize grouping and then adjust *k* to the target risk level. These observations align with the ICCCN 2024 measurements [49] while adding a clearer parameter sensitivity interpretation in terms of *k* and *β*.

To further examine parameter sensitivity, we conducted a focused ablation on the policy parameters λ, c, and γ, and we separately evaluated the entropy-driven adaptive $l_{t}$ controller. All ablations were performed at a representative operating point $\left(k,l,\beta )=(10,2,50\right)$. Each configuration was repeated n=5 times using fixed random seeds, and the same seeds were shared across settings to enable paired comparisons. We report the results as mean ± standard deviation (SD) values across repeated runs. Statistical significance was assessed against the default setting $\left(\lambda ,c,\gamma )=(0.7,3,3\right)$ for the primary metrics (Runtime and IL) using the paired Wilcoxon signed-rank test with per-table Bonferroni correction. Table 10 shows that λ, c, and γ act as interpretable control levers that trade off runtime against distortion (IL) under fixed $\left(k,l,\beta \right)$. Increasing λ strengthens swap-oriented repairs, which tends to improve utility preservation and diversity quality (LSR/entropy) while increasing runtime due to higher search/repair effort. Conversely, decreasing λ biases decisions toward merges, which can reduce runtime but may increase distortion under oversized merges. Increasing c expands the neighbor search space and can stabilize constraint satisfaction at the cost of higher search overhead; in our setting, a small default (c=3) provides a practical balance. The growth cap γ controls the maximum merge size: a larger γ can reduce retries and improve runtime but increases the risk of oversized merges that inflate IL, whereas a tighter γ improves distortion control but may increase swap/retry effort. Overall, γ is best viewed as a balancing knob with an operating-point-dependent optimum between runtime and distortion. Table 11 indicates that enabling adaptive $l_{t}$ reduces unnecessary reconfiguration when SA mixing is favorable (lower ρ), thereby mitigating tail latency (p99) while maintaining strong diversity quality (LSR/entropy). Under more adverse conditions, the controller tightens $l_{t}$ as the skew intensifies to preserve diversity, with bounded overhead. These results support robustness under non-stationery and stress conditions. Taken together, Table 10 and Table 11 provide concise deployment guidance. Under a fixed delay budget β, λ, c, and γ can be tuned to balance responsiveness and utility, and enabling adaptive $l_{t}$ further improves robustness by suppressing excessive reconfiguration and tail latency without aggressive manual retuning. The Δ metrics were computed as (the difference between each setting and the default using paired runs with matched seeds.

Table 11 isolates the impact of enabling the entropy-driven adaptive $l_{t}$ controller under the default operating point. Enabling the controller reduced the reconfiguration frequency (ρ) from 1.45 to 1.10 and improved the tail latency (p99) from 120 ms to 105 ms while keeping IL essentially unchanged (0.60) and preserving diversity quality (LSR/entropy). The throughput also slightly increased (17,200 → 17,800 events/s), indicating that the controller can suppress unnecessary repairs when SA mixing is favorable and tighten $l_{t}$ under adverse skew with bounded overhead. Rows OFF/ON compare the proposed method with the adaptive controller disabled/enabled; differences should be interpreted as (ON–OFF) (no SUHDSA baseline in this table).

### 4.4. Threat Model and Attack Protocol

We consider an external adversary whose goals are (i) identity disclosure (reidentifying a target individual in the released stream) and/or (ii) attribute disclosure (inferring the target’s sensitive attribute (SA), e.g., income bracket). We adopt a prosecutor-style assumption: the attacker knows that the target is present in the released stream and possesses the target’s quasi-identifier (QID) values (e.g., education, occupation, native country) from auxiliary sources. In line with Kerckhoffs’ principle, the attacker is assumed to know the anonymization mechanism and the configuration parameters (*k*, *l*, *β*), as well as the QID generalization/taxonomy used by the publisher. The attacker only has access to released anonymized outputs (released groups or their representatives) and any published group metadata and has no access to raw records, internal buffering states, or intermediate groups prior to release.

To quantify identity disclosure, we implement a conservative record-linkage protocol based on QID compatibility. Given a target QID tuple x, the attacker ranks released candidates by how well their generalized QID values match x. We compute a QID match score using the same taxonomy/containment logic as the anonymization pipeline: a target categorical QID value is treated as compatible with a generalized value if it is contained by the generalized category (or ancestor) induced by the taxonomy. Candidates are then ranked by increasing mismatch/distance score, with deterministic tie-breaking.

We report re-ID@K as the empirical probability that the correct target is contained in the top-K ranked candidates, for K∈{1,5}. In addition, we use the prosecutor-model interpretation of *k*-anonymity: when a target is indistinguishable among at least-k QID-equivalent records in a released group, a QID-only attacker cannot do better than random guessing within that group, yielding an upper-bound reference of approximately 1/k for exact top-1 linkage.

To quantify attribute disclosure, we assume the attacker first identifies the best-matching released group (or representative) using the linkage protocol above and then predicts the target’s SA using the within-group SA distribution. We adopt a simple and reproducible decision rule: the attacker outputs the maximum a posteriori (MAP) SA value, i.e., the most frequent SA value in the matched group. The attribute disclosure rate is defined as the fraction of targets for which this MAP prediction equals the true SA.

Note that (distinct) *l*-diversity guarantees that each released group contains at least l distinct SA values, but it does not, by itself, impose a universal 1/l upper bound on the attacker’s success probability under skewed SA distributions. Accordingly, we report attribute disclosure empirically under the MAP attacker, and we interpret it jointly with the diversity metrics already reported in Section 4.3 (e.g., LSR and SA entropy), which capture the degree of SA mixing within released groups.

We evaluate linkage and attribute inference using the same stream replay setting as in Section 4.3, under a fixed delay budget β, and we report results for representative privacy settings (k,l)∈{(10,2),(20,3)}. Table 12 summarizes re-ID@1, re-ID@5, and the attribute disclosure rate, together with the prosecutor-model *k*-anonymity reference bound 1/k.

Across the tested configurations, re-ID@1 was at or below the prosecutor-model *k*-anonymity reference 1/k: it was 7.9% versus 1/10=10% for $\left(k,l)=(10,2\right)$ and exactly 5.0% versus 1/20=5% for $\left(20,3\right)$ (Table 10). Moreover, attribute disclosure decreased as privacy constraints tightened (38.0% → 24.0%), which is consistent with stronger SA mixing indicated by our diversity metrics, suggesting that enforcing *l*-diversity via dynamic reconfiguration effectively mitigates attribute inference under this threat model.

## 5. Discussion

### 5.1. Interpretation of Findings

The results show that enforcing *l*-diversity in streaming adds a modest runtime overhead relative to SUHDSA while remaining substantially faster than UBDSA (≈45.6% on average). Crucially, we identified operating regions where stronger privacy does not trade off against utility: with moderate–large *k* and a sufficiently wide delay budget *β*, groups tend to include ≥ *l* distinct SA values without heavy transformations, so the reconfiguration frequency falls, and distortion can decrease versus k-only baselines. Specifically, Table 2 reports +4.85–7.87 s of additional runtime at (*k* = 3, *β* = 100) that shrinks to +3.94–4.47 s at (*k* = 50, *β* = 100); Table 3 shows that the utility can be worse by 0.09~0.18 at (*k* = 3, *β* = 10) yet better by 0.03 to 0.19 at (*k* = 10, *β* = 10). These patterns move the narrative from a binary “privacy vs. utility” to a privacy–utility–delay triad, where tuning *k* and *β* exposes sweet spots that deliver stronger protection with acceptable—sometimes improved—utility. Mechanistically, *β* controls the candidate pool within each window (larger *β* → cleaner QID-similar grouping → fewer repairs), and *k* shapes the class composition (larger *k* → higher chance of meeting *l* without costly edits), which together explain the observed declines in both overhead and distortion as (*k*, *β*) increases. The same tendency appears in Table 4: the proposed method’s LSR and H(SA∣G) are consistently higher than those of the baselines across settings, providing numerical evidence that the attribute-disclosure risk is reduced. Under the skewed-SA subset, the divergence widens: *k*-only pipelines frequently emit low-diversity groups (low LSR, zero min-entropy), whereas the proposed method preserves substantially higher LSR and entropy with predictable increases in runtime and IL. This finding supports the claim that diversity-aware, delay-conscious repair is essential when streams exhibit strong class imbalance.

### 5.2. Practical Implications

In deployment, it is important to adopt a tuning sequence that jointly manages delay and risk. Our recommended procedure is straightforward. First, a sufficiently large β (the per-window buffer/delay budget) within the service’s allowable latency is provided, thereby enlarging the candidate pool in each window and stabilizing the QID-similar grouping. Once the demand for repairs diminishes, k (the anonymity level) increases to the target risk level; under a stable grouping regime, the required privacy can be achieved without excessive generalization. This “β first, then k” ordering is particularly effective in skewed-SA domains because it avoids triggering aggressive swap/merge early on and thus better preserves utility.

Fine-grained control during operation is provided by policy parameters. The weight λ balances distortion (ΔIL) against time (ΔRT): a higher λ biases repairs toward swaps when utility preservation is paramount, whereas a lower λ favors merging when responsiveness dominates. The neighbor cap c limits the search cost during reconfiguration by restricting candidates to the top-c nearest neighbors (small defaults such as c=3 worked well in our tests). The growth cap γ enforces ∣G∪H∣≤γk to prevent oversized merges that inflate IL (values around γ≈3 are effective and can be tightened when utility is the priority).

In regulated domains (e.g., healthcare, finance), β should be selected to respect end-to-end service-level objectives (E2E SLOs) (latency, availability, throughput), and privacy controls should align with ISO/IEC 20889 [47]. Practically, tuning β first to satisfy the SLOs, then dialing k, with λ,c,γ as auxiliary levers, tends to expose operating points where LSR and entropy improve together—i.e., disclosure risk and distortion decline simultaneously (consistent with the patterns in Section 4.3, Table 4). When privacy requirements tighten (higher l), increases in runtime and IL should be anticipated (e.g., at (k,β)=(10,50), average time 7.92→8.41→9.16 s; IL 47.50→52.00→64.50 s). These costs can be offset by enlarging β to expand the candidate pool or by moderating k to curb distortion. Under heavy SA skew, securing the headroom in β is especially effective: it suppresses ρ (repair frequency) while maintaining high LSR and entropy.

Blueprint for higher-dimensional settings: Because our evaluation centers on relatively low-dimensional QIDs (e.g., UCI ADULT [48]), we outline practical steps for scale-up. For numeric QIDs, utility-preserving PCA/autoencoders are applied; for categorical QIDs, taxonomy upleveling with feature hashing/cardinality pruning is used. In distributed execution, partitioning via generalized QID keys, restricting candidates via LSH/blocking, and within partitions combine top-c neighbor searches with ∣G∪H∣≤γk. Accelerate search via ANN indices, batched reconfiguration, and early stopping when the objective λΔIL+(1−λ)ΔRT exhibits diminishing returns. Coupled with our priority-based repair policy, these measures are expected to suppress tail latency (p99) and keep p95 within the service-time target $T_{\beta }=\beta /r$. A comprehensive high-dimensional empirical study is left for future work.

Dynamic control and sensitivity: With fixed l, the reconfiguration frequency ρ (merges + swaps per window·s) can adversely affect tail latency (p99). We therefore employ an entropy-driven adaptive $l_{t}$ controller that relaxes $l_{t}$ when diversity is high and tightens it when skew intensifies, reducing unnecessary reconfigurations while preserving diversity quality. To systematically quantify how reconfiguration affects latency, we propose a runtime-sensitivity methodology. We define ρ as the number of merges + swaps per window·s, partition observations into ρ regimes, and, within each regime, consistently collect (ρ, p50/p95/p99, throughput). We summarize joint behavior with descriptive statistics, test monotonic/linear associations via Spearman/Pearson correlations, and apply piecewise linear regression to estimate marginal effects (e.g., ∂p95/∂ρ). To identify control effects, we repeat the same procedure with the adaptive $l_{t}$ controller ON/OFF and assess whether p99 becomes less sensitive to ρ. This procedure serves as a methodological blueprint; detailed execution protocols are given in Appendix A.

Fair assessment of downstream utility: We evaluate the impact of anonymization on analytics under a fair, matched setting—raw data vs. anonymized data (ours and baselines), with identical models, tuning, and data splits. The task suite comprises classification (AUROC, F1—emphasizing minority class F1), correlation/regression (Pearson/Spearman correlations, MI, RMSE, MAE), streaming anomaly detection (precision@k, ingress → sink alert delay), and clustering (ARI, NMI, silhouette). All tasks are run for n=5 repetitions, and we report the median relative performance vs. raw performance with confidence intervals.

External validity and statistical power: Sensitivity estimates are contingent on interactions among hardware configurations, workload patterns (skew, bursts, drift, missingness), tuning parameters (β,c,γ,λ), and dataset characteristics (QID dimensionality and category granularity). Ensuring external validity and adequate power therefore requires factorial, large-scale experiments spanning multiple datasets, hardware setups, and workload scenarios. Accordingly, this paper first establishes standardized evaluation procedures and metric definitions, together with reproducible methods/protocols (Appendix A), to enable replication and extension by the community; comprehensive quantitative comparisons will be reported in follow-up work adopting this expanded experimental design.

Relation to complementary models: Finally, *t*-closeness focuses on distributional closeness under a threat/runtime model different from our real-time k/l enforcement with dynamic reconfiguration, so it is excluded from the current baselines; we plan a dedicated extension that integrates a closeness constraint into reconfiguration. Differential privacy (DP) is complementary to our pipeline: DP post-processing of released representatives/SA histograms and DP microaggregation, together with per-window/slide privacy accounting (composition rules), can strengthen resilience to linkage/background-knowledge attacks (with utility trade-offs to be quantified). During deployment, adaptive control helps keep p95 within the target while balancing the IL and the risk bound (approximately 1/k+1/l). The $l_{t}$ controller reacts to changes in SA skew; error-driven tuning of β stabilizes latency under workload shifts, and k adjustments—guarded by hysteresis, minimum dwell time, and parameter bounds—avoid oscillations while trading off between the IL and the theoretical risk bound (approximately 1/k+1/l). For more aggressive settings, a lightweight bandit/RL scheme could optimize a reward R, although this requires additional tuning and cost and is left to future work. The same tuning principles extend to high-skew, bursty streams (e.g., IoT/biomedical); Section 4.1 provides a procedural external validity check via synthetic scenarios, and the synthetic-workload blueprint in Section 4.1/Section 4.3 serves as a lightweight surrogate for probing generalization to streams with skew, bursts, and drift.

### 5.3. Limitations, Threats to Validity, and Mitigation Strategies

- Dataset and generality: Our results are based on the UCI Adult. (Mitigation: use the synthetic-workload blueprint at the end of Section 4.1/Section 4.3—Zipf/exponential/mixture skew, bursts, drift, missingness—to emulate domain characteristics and strengthen external validity with multiple real datasets such as sensor/biomedical telemetry.)
- High dimensionality (*m*): Higher-dimensional QIDs increase the grouping/microaggregation cost and p95/p99 latency. (Mitigation: apply the Section 5.2 mitigation blueprint—dimensionality reduction (PCA/AE), categorical uplevel/feature hashing, distributed partitioning (generalized QID keys, locality-sensitive hashing (LSH)/blocking), approximate nearest neighbor (ANN)-based search acceleration, and batched reconfiguration/early stopping) to suppress p99 tails and keep p95 ≤ *T_β_*.)
- Heavy skew/adversarial distributions: A surge in violators (ν) can push reconfiguration toward conservative bounds. (Mitigation: increase *β* within SLOs; adjust λ downward or γ upward to cut retries; introduce SA-aware seeding.)
- Heuristic choices: Distance metrics and the λ-weighted rule are reasonable but not unique. (Mitigation: evaluate learned or taxonomy-aware metrics; ablate *λ*, *c*, and *γ* for robustness.)
- Single-node implementation: The measurements reflect an optimized single-machine Python code path. (Mitigation: prototype distributed execution in Flink/Spark; colocation of QID-similar partitions.) We do not report end-to-end measurements from a live Kafka/Flink cluster deployment in this paper.

To reduce internal validity threats, we applied the same *δ* adaptation and CAIL base across all methods and swept comparable (*k*, *β*) grids under a fixed environment; nevertheless, broader datasets and distributed runs would strengthen external validity.

### 5.4. Future Work Directions

The findings point to several extensions: (i) parallel/distributed grouping and reconfiguration to preserve responsiveness at higher rates and dimensionalities; (ii) data-aware or learned similarity to reduce the repair frequency, especially for large m; (iii) online control that adapts (*λ*, *c*, *γ*) from the observed ΔIL and ΔRT under drift; (iv) selective layering of stronger models (e.g., *t*-closeness) on high-risk groups; and (v) robustness and fairness analyses to ensure that repairs do not disproportionately impact subpopulations. In summary, real-time l-diversity is feasible with modest overhead, and with the right operating point, stronger privacy can coincide with stable or improved utility—a practical takeaway for delay-constrained streaming systems. Our E2E delay results are simulation-based on UCI Adult [48] and do not include live network jitter or cluster-level scheduling effects; they are intended to complement the runtime integration blueprint (Section 3) rather than substitute for a full-scale deployment study, which we leave for future work.

## 6. Conclusions

This work extends SUHDSA with real-time *l*-diversity enforcement—a lightweight monitor–trigger–repair mechanism that verifies SA diversity during grouping and, upon violation, performs swap/merge before microaggregation. By embedding *l*-diversity into a high-throughput *k*-anonymity pipeline, the method addresses SUHDSA’s vulnerability to attribute skew while preserving streaming feasibility.

Experiments on UCI Adult show a modest runtime increase over SUHDSA—+3.94–7.87 s depending on (*k*, *β*)—yet the method remains ≈45.6% faster than UBDSA. Utility effects depend on the operating point: with low *k* and tight β, IL can increase (up to +0.18 absolute vs. SUHDSA), whereas with larger *k* or broader *β*, it can decrease (down to −0.19), revealing privacy–utility–delay regimes where stronger privacy also reduces distortion. These findings confirm that real-time l-diversity is practical and can improve data quality under appropriate parameterization. Importantly, quantitative privacy metrics (LSR and entropy) empirically substantiate the reduction in attribute-disclosure risk beyond the theoretical argument. The experiments fix QIDs to (education, occupation, native country), enforce *k* on this tuple, and vary l ∈ {2,3,5} by discretizing/synthetically assigning income brackets, isolating the specific impact of diversity strength. Additionally, an explicit l sweep at fixed (k,β)=(10,50) confirms monotonic effects: the average runtime is 7.92 → 8.41 → 9.16 s, and the average IL is 47.50 → 52.00 → 64.50 for l=2,3,5, whereas the *k*-only baselines remain unchanged across l. On a skewed-SA subset (≈0.85 majority), we observe the same monotonic trade-offs—higher LSR/entropy and predictable increases in runtime and IL—while *k*-only baselines suffer dramatically lower LSR and zero min-entropy cases, reinforcing the method’s advantage under adverse class imbalance.

The contributions of this study are threefold: (i) A streaming mechanism that jointly enforces *k*-anonymity and *l*-diversity (aligned with ISO/IEC 20889 [47]) using dynamic reconfiguration with auditable release checks (|*G*| ≥ *k*, *D*(*G*) ≥ *l*); (ii) an empirical characterization of sweet spots in *(k*, *β)*; where runtime remains low and utility is preserved or improved, clarifying when l-diversity helps rather than harms; (iii) practical guidance for deployment—increase *β* first within end-to-end service-level objectives and then set *k* to the target risk level, using *λ*, *c*, and *γ* as tunable parameters to balance the utility (ΔIL) and runtime (ΔRT).

Limitations include sensitivity to high QID dimensionality and extreme SA skew, where the rate of diversity violations can increase the reconfiguration cost. Nonetheless, the observed overheads are small in typical regimes, and the method consistently outperforms UBDSA in terms of runtime while closing SUHDSA’s attribute-disclosure gap.

Future work will explore distributed execution for grouping and reconfiguration, learned or taxonomy-aware similarity to cut repairs, online control of (*λ*, *c*, *γ*) under drift, and selective layering of *t*-closeness on high-risk groups. Together, these directions aim to harden privacy guarantees while maintaining the responsiveness demanded by real-time analytics in sensing, finance, healthcare, and large-scale online services.

## Figures and Tables

**Table 1:** Experimental settings for the dataset, parameters, and window/delay rules.

Item	Configuration/Description	Note
Dataset	UCI ADULT (Census Income) [48], 32,561 tuples, 14 attributes	Categorical examples: Education, Occupation, Native Country
QID	Education, Occupation, Native country	Fixed across all runs
SA	Income; *l* = 2: binary (≤50 K/>50 K); *l* = 3: 3 brackets (≤30, (30,60], >60); *l* = 5: 5 brackets (≤20, (20,40], (40,60], (60,80], >80); multibracket values are synthetically assigned; QIDs unchanged	Used to isolate the effect of *l*
Implementation/Environment	Python (common code base), Windows 11, i9-12900KS 3.40 GHz, RAM 64 GB	Same environment family as ICCCN’24 [49]
Window/Buffer	FIFO buffer, *β*: maximum allowed delay (maximum number of tuples held simultaneously)	*β* ∈ {10, 50, 100}
Parameter Grid	*k* ∈ {3, 10, 50}, *β* ∈ {10, 50, 100}, *l* ∈ {2, 3, 5}	
Delay Threshold δ Rule	Adaptive δ adjustment based on UBDSA joint minimization of average delay and IL	Applied equally to SUHDSA [4] and proposed method
UBDSA [25] (our reimplementation)	*k*-anonymity, CAIL distance, no *l*-diversity	*δ* adaptive adjustment
SUHDSA [4] (our reimplementation)	*k*-anonymity, CAIL-based fast aggregation/optimization, no *l*-diversity	*δ* adaptive adjustment (same as UBDSA)
Proposed Method (this work)	*k*-anonymity and *l*-diversity verification, dynamic reconfiguration (merge/swap), CAIL retained	*δ* adaptive adjustment (same)

**Table 2:** Runtime (s) under varying k and β on the UCI ADULT stream replay (*n* = 5 repetitions; fixed random seeds shared across methods; mean ± SD and 95% CI over repeated runs; the proposed algorithm vs. SUHDSA compared using the paired Wilcoxon signed-rank test with Bonferroni correction).

Condition	Algorithm	Runtime (s), Mean ± SD	95% CI (Mean)	Runtime, Min–Max	ΔRuntime (Proposed–SUHDSA), Mean	*p*-Value † (Corr.)
*k* = 3, *β* = 100	Proposed Algorithm (A)	12.30 ± 2.10	[9.70, 14.90]	9.22–15.54	+6.25	0.125
	SUHDSA (B)	6.05 ± 1.10	[4.69, 7.41]	4.37–7.67	-	-
	UBDSA	33.70 ± 0.30	[33.33, 34.07]	33.24–34.15	-	-
*k* = 50, *β* = 100	Proposed Algorithm (A)	10.05 ± 1.10	[8.69, 11.41]	8.43–11.92	+4.10	0.250
	SUHDSA (B)	5.95 ± 1.05	[4.65, 7.25]	4.49–7.45	-	-
	UBDSA	32.80 ± 0.15	[32.61, 32.99]	32.64–32.95	-	-

† Wilcoxon signed-rank test (paired, *n* = 5); Bonferroni-corrected (*m* = 2 conditions in this table).

**Table 3:** IL (0–1) under varying k and β on the UCI ADULT stream replay (*n* = 5 repetitions; fixed random seeds shared across methods; mean ± SD and 95% CI over repeated runs; the proposed algorithm vs. SUHDSA compared using the paired Wilcoxon signed-rank test with Bonferroni correction).

Condition	Algorithm	IL, Mean ± SD	95% CI (Mean)	IL, Min–Max	ΔIL (Proposed–SUHDSA), Mean	*p*-Value † (Corr.)
*k* = 3, *β* = 10	Proposed Algorithm (A)	0.62 ± 0.04	[0.57, 0.67]	0.54–0.70	+0.13	0.125
	SUHDSA (B)	0.49 ± 0.08	[0.39, 0.59]	0.36–0.61	-	-
	UBDSA	0.98 ± 0.00	[0.98, 0.98]	0.98–0.98	-	-
*k* = 10, *β* = 10	Proposed Algorithm (A)	0.64 ± 0.06	[0.57, 0.71]	0.55–0.75	+0.06	0.250
	SUHDSA (B)	0.58 ± 0.11	[0.44, 0.72]	0.36–0.72	-	-
	UBDSA	0.98 ± 0.00	[0.98, 0.98]	0.98–0.98	-	-

† Wilcoxon signed-rank test (paired, *n* = 5); Bonferroni-corrected (*m* = 2 conditions in this table).

**Table 4:** Quantitative privacy metrics under varying *k* and *β* on UCI ADULT (SA = income, binary; log base = 2).

Condition(k,β,l)	Algorithm	LSR (%)	H(SA|G) Mean (Bits)	H(SA|G) Min (Bits)
(3, 10, 2)	Proposed (*k* + *l*, reconfig)	96	0.78	0.32
	SUHDSA (*k*-only)	61	0.55	0.00
	UBDSA (*k*-only)	48	0.49	0.00
(10, 10, 2)	Proposed (*k* + *l*, reconfig)	99	0.85	0.42
	SUHDSA (*k*-only)	75	0.62	0.00
	UBDSA (*k*-only)	58	0.52	0.00
(3, 100, 2)	Proposed (*k* + *l*, reconfig)	98	0.84	0.38
	SUHDSA (*k*-only)	70	0.60	0.00
	UBDSA (*k*-only)	55	0.52	0.00
(50, 100, 2)	Proposed (*k* + *l*, reconfig)	100	0.90	0.55
	SUHDSA (*k*-only)	88	0.72	0.00
	UBDSA (*k*-only)	70	0.60	0.00

**Table 5:** Baselines (fixed across l) and l sweep for the proposed method at fixed (k,β)=(10,50).

Block	*l*	Algorithm	Runtime (s), Avg	IL (0–1), Avg
Baselines	–	SUHDSA (k-only)	6.17	0.46
	–	UBDSA (k-only)	33.14	0.99
Proposed (*l* sweep)	2	Proposed (*k* + *l*, reconfig)	7.92	0.48
	3		8.41	0.52
	5		9.16	0.64

**Table 6:** Validation on the skewed-SA subset (majority ratio ρ≈0.8–0.9).

Block	*l*	Algorithm	Runtime (s), Avg	IL (0–1), Avg	LSR (%)	H(SA|G) Mean (Bits)	H(SA|G) Min (Bits)
Baselines	–	SUHDSA (k-only)	6.17	0.46	12	0.95	0.20
	2	Proposed (*k* + *l*, reconfig)	8.30	0.50	92	0.82	0.45
Proposed (*l* sweep)	3	Proposed (*k* + *l*, reconfig)	8.95	0.55	80	1.30	0.88
	5		9.90	0.69	62	1.75	1.05

**Table 7:** End-to-end (E2E) delay (simulation) on UCI ADULT [48] (p95 target: *T_β_ = β/r* (s); r: mean arrival rate (= Throughput in this table).

Mode	Throughput (Events/s)	p50 (ms)	p95 (ms)	p99 (ms)	Notes
Processing-only (δ=0 ms)	20,000	18	35	60	Ingress → sink (including grouping/reconfiguration; excludes physical network)
+Synthetic network (δ=2 ms)	19,000	20	38	65	Ingress → sink including +2 ms/record
+Synthetic network (δ=5 ms)	17,500	25	45	75	Ingress → sink including +5 ms/record

**Table 8:** Synthetic stress tests (skew/bursts/drift): utility and privacy (no missingness; (*k*,*l*,*β*) = (10,2,50); mean ± SD over *n* = 5 repetitions; fixed random seeds shared across methods).

Scenario	Method	Runtime (s)	IL (0–1)	LSR (%)	Entropy *H*(SA|*G*) Mean (Bits)
Skew (SA majority ratio ≈ 0.85)	SUHDSA (*k*-only)	6.25 ± 0.18	0.47 ± 0.01	12 ± 4	0.28 ± 0.06
	Proposed (*k* + *l*, reconfig)	8.35 ± 0.26	0.50 ± 0.02	92 ± 3	0.82 ± 0.05
Bursts (arrival spike ×5)	SUHDSA (*k*-only)	6.40 ± 0.20	0.48 ± 0.01	55 ± 7	0.50 ± 0.06
	Proposed (*k* + *l*, reconfig)	8.70 ± 0.32	0.51 ± 0.02	95 ± 2	0.84 ± 0.04
Drift (strong, gradual drift)	SUHDSA (*k*-only)	6.35 ± 0.19	0.48 ± 0.01	45 ± 8	0.46 ± 0.07
	Proposed (*k* + l, reconfig)	8.55 ± 0.30	0.51 ± 0.02	94 ± 2	0.83 ± 0.05

**Table 9:** Synthetic stress tests (skew/bursts/drift): E2E latency, throughput, and reconfiguration frequency ρ (no missingness; (*k*,*l*,*β*) = (10,2,50); mean ± SD over *n* = 5 repetitions; fixed random seeds shared across methods).

Scenario	Method	Throughput (Events/s)	p50 (ms)	p95 (ms)	p99 (ms)	*ρ* (Reconfigs/s)
Skew (≈0.85)	SUHDSA	20,400 ± 700	18 ± 1	36 ± 2	65 ± 5	0.00
	Proposed	18,100 ± 800	20 ± 1	46 ± 3	95 ± 8	1.40 ± 0.20
Bursts (×5)	SUHDSA	19,200 ± 900	19 ± 1	44 ± 4	85 ± 10	0.00
	Proposed	16,200 ± 1000	23 ± 2	62 ± 6	140 ± 15	1.85 ± 0.25
Drift (strong)	SUHDSA	19,600 ± 800	19 ± 1	42 ± 3	78 ± 9	0.00
	Proposed	17,100 ± 900	22 ± 2	54 ± 5	112 ± 12	1.30 ± 0.20

**Table 10:** Ablation on λ, c, and γ at $\left(k,l,\beta )=(10,\;2,\;50\right)$ (default: λ=0.7, c=3, γ=3; *n* = 5; mean ± SD; fixed random seeds).

Factor	Setting	Runtime (s) Mean ± SD	IL Mean ± SD	LSR Mean ± SD	Entropy Mean ± SD	ΔRuntime (Setting–Default)	*p* † (RT)	ΔIL (Setting–Default)	*p* † (IL)
–	Default	8.40 ± 0.30	0.60 ± 0.03	99 ± 1	0.86 ± 0.03	0.00	–	0.000	–
λ	0.3	7.90 ± 0.25	0.64 ± 0.03	98 ± 1	0.84 ± 0.03	−0.50	0.250	+0.040	0.250
	0.5	8.10 ± 0.28	0.62 ± 0.03	98 ± 1	0.85 ± 0.03	−0.30	0.500	+0.020	0.500
	0.9	8.85 ± 0.35	0.58 ± 0.03	99 ± 1	0.87 ± 0.03	+0.45	0.250	−0.020	0.250
c	1	8.05 ± 0.27	0.63 ± 0.03	97 ± 2	0.83 ± 0.04	−0.35	0.500	+0.030	0.500
	5	9.10 ± 0.40	0.59 ± 0.03	99 ± 1	0.87 ± 0.03	+0.70	0.125	−0.010	1.000
γ	2	8.95 ± 0.35	0.58 ± 0.03	99 ± 1	0.87 ± 0.03	+0.55	0.250	−0.020	0.250
	4	8.10 ± 0.30	0.63 ± 0.03	98 ± 1	0.85 ± 0.03	−0.30	0.500	+0.030	0.500

† Paired Wilcoxon signed-rank test vs. default; Bonferroni-corrected within this table.

**Table 11:** Effect of the adaptive $l_{t}$ controller at $\left(k,\;l,\;\beta )=(10,\;2,\;50\right)$ under the default: λ=0.7, c=3, γ=3; *n* = 5; mean ± SD; fixed random seeds).

$l_{t}$	Runtime (s) Mean ± SD	IL Mean ± SD	LSR Mean ± SD	Entropy Mean ± SD	p95 (ms) Mean ± SD	p99 (ms) Mean ± SD	Throughput Mean ± SD	ρ Mean ± SD	*p* † (p99)	p † (ρ)
OFF	8.40 ± 0.30	0.60 ± 0.03	99 ± 1	0.86 ± 0.03	55 ± 5	120 ± 12	17,200 ± 900	1.45 ± 0.20	–	–
ON	8.25 ± 0.28	0.60 ± 0.03	98 ± 1	0.85 ± 0.03	50 ± 4	105 ± 10	17,800 ± 950	1.10 ± 0.18	0.250	0.250

† Paired Wilcoxon signed-rank test (ON vs. OFF); Bonferroni-corrected within this table.

**Table 12:** Linkage and attribute inference under the prosecutor threat model.

(*k*, *l*)	Re-ID@1 (%)	Re-ID@5 (%)	Attribute Disclosure (%)	(1/*k*) Reference (%)
(10, 2)	7.9	25.0	38.0	10.0
(20, 3)	5.0	18.0	24.0	5.0

## Data Availability

The original data presented in the study are openly available in UCI Machine Learning Repository Adult at https://archive.ics.uci.edu/dataset/2/adult (accessed on 1 November 2025).

## Appendix A. Runtime Sensitivity Protocol

### Appendix A.1. Objective and Scope

This appendix specifies a reproducible procedure to quantify the relationship between the reconfiguration frequency ρ (merges + swaps per window·s) and latency (p50/p95/p99)/throughput (events/s) and to assess whether the adaptive $l_{t}$ controller (Section 4.3) can mitigate tail latency (p99) sensitivity to ρ. It is methodological; no quantitative results are reported here.

### Appendix A.2. Metrics and Notations

- ρ*: reconfiguration frequency*.

(A1)
$$\;\;\;\;\;\;\;\;\rho_{w}=\sum 1\left\{\mathrm{swap}\;\mathrm{or}\;\mathrm{merge}\;\mathrm{in}\;\mathrm{window}\;w\right\},\;\rho_{s}=\sum_{t\in s}1\{\mathrm{swap}\;\mathrm{or}\;\mathrm{merge}\;\mathrm{at}\;t\}$$

- Latencies: p50, p95, p99 (ms)—measured ingress → sink; processing-only or with synthetic per-record delay δ.
- Throughput: events/s.
- Controller: $l_{t}$ (optional; default OFF), using EMA/hysteresis/dwell as in Section 3.2.

### Appendix A.3. Experimental Design

- Dataset/engine: Same as in Section 4 (pipeline, hardware/software).
- Factors (factorial example):
  ■ input skew $\in \left\{\mathrm{low},\;\mathrm{mid},\;\mathrm{high}\right\};$
  ■ β∈{10,50,100} (cardinality);
  ■ c∈{2,3} (nearest neighbors);
  ■ (opt.) Synthetic delay δ∈{0,2,5} ms/record.
- Repetitions/Length: 5 repetitions per condition; 30 s windows (or N windows).
- ON/OFF comparison: Run with the controller OFF and ON under the same design.

### Appendix A.4. Instrumentation

- ρ logging: reset per window; increment by 1 on each swap/merge (see Algorithm 1 lines 32/35).
- Latency/Throughput: aggregate p50/p95/p99 and throughput per window.
- Metadata: factors (skew, β, c, δ), seeds, and failed/dropped windows.

### Appendix A.5. Preprocessing

- Validity rules: exclude failed/dropped windows by predefined criteria (record reasons).
- Aggregation: report median/IQR or 95% CIs per condition.
- Normalization (opt.): express p95 relative to $T_{\beta }=\beta /r$ (r = Throughput) for comparability.

### Appendix A.6. Analysis

1. Correlations
  - Spearman (monotonic) and Pearson (linear) correlations between ρ and p95/p99/throughput (α=0.05).
2. Piecewise regression
  - Fit p95=a+b ρ by segments to estimate the slope b=∂p95/∂ρ (repeat for p99 and throughput).
  - Robustness: bootstrap (≥1000 resamples) CIs for b; Huber/quantile regression checks.
3. ON/OFF comparison
  - b is compared across OFF vs. ON, and the slope-reduction ratio $(b_{\mathrm{OFF}}-b_{\mathrm{ON}})/b_{\mathrm{OFF}}$ is reported.
  - Do the same for p99 (tail sensitivity).
4. Reporting rule
  - Interpret as a methodological comparison (no numeric results in this paper).

### Appendix A.7. Reporting Checklist

- Per-condition summary: *ρ*, p50/p95/p99, throughput (median [IQR] or 95% CI).
- Correlation summary: Spearman/Pearson correlations and significance.
- Piecewise sensitivity summary: piecewise slopes *b* with 95% CIs (OFF/ON; p95/p99/throughput).
- Optional visual diagnostics: scatter plots of *ρ* vs. p95 and *ρ* vs. p99 with fitted lines (OFF/ON overlaid).

**Note:** This appendix provides reproducibility-oriented reporting guidance; no actual result tables/figures are included.

### Appendix A.8. Reproducibility (Recommended)

- Environment: OS/runtime/library versions; CPU/GPU/memory.
- Seeds: random seeds.
- Config: all factor settings (skew, β, c, δ); controller parameters (α, hysteresis band, dwell).

## Appendix B. Entropy-Driven Adaptive $l_{t}$ Controller (Pseudocode)

This appendix summarizes the optional entropy-driven adaptive $l_{t}$ controller referenced in Section 3.2 and Algorithm 1. The controller estimates SA mixing in the current buffer via entropy, smooths it with EMA, and adjusts $l_{t}$ within $\left[l_{min},l_{max}\right]$. To avoid oscillation, it applies a hysteresis band and a minimum dwell time.

**Definitions.** Let p(v) be the empirical frequency of SA value v in the current buffer $Buf_{t}$. The SA entropy is

$$H_{t}=U-\sum_{v}p\left(v\right)\log p\left(v\right).$$

EMA smoothing is ${\hat{H}}_{t}=\alpha H_{t}+(1-\alpha ){\hat{H}}_{t-1}$. Normalized entropy is $e_{t}=clip\;({\hat{H}}_{t}/log|S|,0,1).$

**Algorithm A1**: AdaptiveLtController(*Buf_t*, *l_min*, *l_max*, *α*, HysteresisBand, DwellWindows, Hhat_prev, e_prev, lt_prev, last_change_t, |*S*|)
1 *H_t* ← Entropy(*Buf_t.S*) // as defined above 2 *Hhat_t ← α·H_t* + (1−*α*)·Hhat_prev 3 *e_t ←* clip(*Hhat_t* / log(|*S*|), 0, 1) 4 *l_t__cand* ← floor(*l_min + (l_max − l_min)·e_t*) 5 // floor: enforce integer lt (diversity level is discrete) 6 // or (*1−e_t*), keep consistent with Algorithm 1 7 *l_t__cand ←* clip(*l_t__cand, l_min, l_max*) 8 // clip: bound lt within [l_min, l_max] to avoid overly strict/loose targets 9 if (*t − last_change_t*) < DwellWindows then 10 *l_t_ ← l_t__prev* 11 else if |*e_t − e_prev*| < HysteresisBand then 12 *l_t_ ← l_t__prev* 13 else 14 *l_t_ ← l_t__cand*; *last_change_t ← t* 15 end if 16 return (*l_t_, Hhat_t, e_t, last_change_t*)
